# Integrating Gene Expression Data Into Genomic Prediction

**DOI:** 10.3389/fgene.2019.00126

**Published:** 2019-02-25

**Authors:** Zhengcao Li, Ning Gao, Johannes W. R. Martini, Henner Simianer

**Affiliations:** ^1^Animal Breeding and Genetics Group, Department of Animal Sciences, Center for Integrated Breeding Research, University of Göttingen, Göttingen, Germany; ^2^State Key Laboratory of Biocontrol, Guangzhou Higher Education Mega Center, School of Life Science, Sun Yat-sen University, Guangzhou, China; ^3^KWS SAAT SE, Einbeck, Germany

**Keywords:** GRBLUP, transcriptome, phenotype prediction, *Drosophila melanogaster*, epistasis

## Abstract

Gene expression profiles potentially hold valuable information for the prediction of breeding values and phenotypes. In this study, the utility of transcriptome data for phenotype prediction was tested with 185 inbred lines of *Drosophila melanogaster* for nine traits in two sexes. We incorporated the transcriptome data into genomic prediction via two methods: GTBLUP and GRBLUP, both combining single nucleotide polymorphisms (SNPs) and transcriptome data. The genotypic data was used to construct the common additive genomic relationship, which was used in genomic best linear unbiased prediction (GBLUP) or jointly in a linear mixed model with a transcriptome-based linear kernel (GTBLUP), or with a transcriptome-based Gaussian kernel (GRBLUP). We studied the predictive ability of the models and discuss a concept of “omics-augmented broad sense heritability” for the multi-omics era. For most traits, GRBLUP and GBLUP provided similar predictive abilities, but GRBLUP explained more of the phenotypic variance. There was only one trait (olfactory perception to Ethyl Butyrate in females) in which the predictive ability of GRBLUP (0.23) was significantly higher than the predictive ability of GBLUP (0.21). Our results suggest that accounting for transcriptome data has the potential to improve genomic predictions if transcriptome data can be included on a larger scale.

## Introduction

Prediction of genetic values has been a key problem in quantitative genetics. Since Meuwissen et al. (2001) published the landmark article, which uses whole genome single nucleotide polymorphisms (SNPs) to modify the traditional prediction of breeding values using family relationship, the concept of “genomic selection” has revolutionized animal and plant breeding. A number of statistical approaches have been applied in practice, such as genomic best linear unbiased prediction (GBLUP) (VanRaden, 2008), ridge regression (Whittaker et al., 1999), or the “Bayesian Alphabet” (Gianola et al., 2009; Gianola, 2013). These approaches utilizing genome-wide SNP data have been used to increase the genetic progress of breeding programs by increasing predictive accuracy of breeding values, reducing generation intervals or shortening the breeding cycles. In plant line breeding, genomic prediction focuses on breeding values in early generations of a breeding program, while the genomic prediction of phenotypes may be attractive when estimating the commercial value of cultivars (Crossa et al., 2017). Broad sense heritability is the relevant genetic parameter for phenotypic prediction, which is defined as the ratio of genetic variance over the phenotypic variance. It reflects all genetic contributions to a population's phenotypic variance including additive and non-additive effects such as dominance and epistasis. It was demonstrated that epistasis explains noticeable fractions of variation in human gene expression (Brown et al., 2014). One of the critically important issues for phenotypic prediction and the estimation of broad sense heritability is how to model non-additive effects. There is plenty of literature illustrating an improved prediction of phenotypes when using non-additive relationships (Crossa et al., 2010; Martini et al., 2016; Forsberg et al., 2017; Gao et al., 2017). However, epistatic effects can arise from various interactions between alleles or genotypes at different loci. For more than two genes, higher order interactions may be included, which makes the estimation of epistatic effects very difficult by using typically parametric regression methods. Another problem for the prediction of phenotypes is that from DNA sequences to phenotypes there are complex biological processes that may affect the phenotypes. Even with complete whole sequence information, genomic prediction may not capture multiple interactions between genes and downstream in the biological regulation. The inclusion of additional layers of omics data in the prediction machinery may provide a partial solution for this problem, since for instance transcriptome data may be “closer” to the phenotype, and since an epistatic interaction on the genotype level may be captured by an additive effect on -for instance- the transcriptome level. In the context of defining the respective broad sense heritability for the combination of genotypic data and omics data, the classical concept only covers the proportion of genetic factors including additive or dominance effects and interactions (Lush, 1940). We discuss the concept of “omics-augmented broad sense heritability” to be used in the context of the prediction of phenotypes not only based on effects at the genome level, but also accounting for effects of downstream biological regulation captured by omics data.

Recently, several studies have proposed to exploit transcriptome data as explanatory variables for prediction of traits. Other than nuclear DNA-based SNP data, gene expression levels are affected by several factors, like choice of tissue, time of sampling and experimental conditions, and using only gene expression data in prediction of phenotypes may not be as robust as using SNP markers. Utilizing both genomic marker information and gene expression data could be a promising option. Modeling gene expression data as a predictor into genomic prediction is expected to explain more epistatic variance or complex biological regulation processes and potentially increases predictive accuracy. González-Reymúndez et al. (2017) integrated whole-omics data (including whole-genome gene expression profiles) into breast cancer prediction, and demonstrated that omics and omic-by-treatment interactions explain a sizable fraction of the variance of survival time, and further suggested that whole-omic profiles could be used to improve prognosis prediction accuracy among breast cancer patients. Guo et al. (2016) showed that gene expression levels provided reduced predictive abilities compared to those based on genetic markers. When combing gene expression data with SNPs, the predictive abilities are either greater than or comparable to those with GBLUP alone. When comparing marker genotype to gene expression data to predict resistance of soybean plants to the pathogen *Phytophthora sojae*, Loh et al. (2011) found that the latter performed better than genotype markers alone. Zarringhalam et al. (2018) obtained robust phenotype predictions from gene expression data using differential shrinkage of co-regulated genes. Kang et al. (2017) developed a biological network-based regularized artificial neural network model for prediction of phenotypes from transcriptomic measurements in clinical trials, which significantly improved the robustness and generalizability of predictions to independent datasets. Moreover, different types of omics data have been used for hybrid prediction in Maize (Westhues et al., 2017; Schrag et al., 2018).

Reproducing kernel Hilbert space regression (RKHS), a semi-parametric prediction method, was introduced by Gianola et al. (2006) to the field of animal breeding. It was promoted as an alternative option to capture the complicated interactions between genes. Jiang and Reif (2015) illustrated that the Gaussian kernel models interaction effects implicitly. More importantly, RKHS provides a simple framework to incorporate information on pedigrees, markers, or any other form of data characterizing the genetic background of individuals (de los Campos et al., 2009). Hu et al. (2015) used RKHS for evaluating the utility of methylation information in prediction of plant height, and demonstrated that epigenetic variation accounted for 65% of the phenotypic variance. In the present study, we used five kernel-based methods: GBLUP, TBLUP, RKHS, GTBLUP, and GRBLUP. Genomic best linear unbiased prediction (GBLUP) using SNP data is set to be a benchmark model. TBLUP and RKHS are used for transcriptomic prediction, where the first uses a linear kernel and the latter uses a Gaussian kernel. Moreover, we define GTBLUP (combining GBLUP and TBLUP) and GRBLUP (combining GBLUP and RKHS) utilizing both transcriptome data and whole-genome sequence data.

*Drosophila melanogaster* is a widely used model organism for biological research in genetics, physiology, microbial pathogenesis, and life history evolution, and it has been demonstrated that the architecture of *Drosophila* quantitative traits is dominated by extensive epistasis (Huang et al., 2012). Making use of *Drosophila* omics data stands a chance to capture the prevalent epistasis for phenotype prediction. The *Drosophila melanogaster* Genetic Reference Panel (DGRP) is a community resource for analysis of population genomics and quantitative traits. It consists of more than 200 fully sequenced inbred lines derived from the Raleigh population, USA (Mackay et al., 2012). We used whole-genome SNP data and gene expression data of 185 Drosophila inbred lines from DGRP in this study. The objective was (1) to combine transcriptome data with whole-genome sequence data for genomic-transcriptomic prediction using GTBLUP and GRBLUP, (2) to assess whether GTBLUP and GRBLUP can capture substantial proportions of phenotypic variances explained by transcriptome data, and (3) to test whether accounting for transcriptome data can improve phenotype prediction.

## Materials and Methods

### Data

#### Whole-Genome Sequence Data

The *Drosophila melanogaster* Genetic Reference Panel (DGRP) is a community resource for analysis of population genomics and quantitative traits. It consists of 205 fully sequenced inbred lines derived from 20 generations of full sibling inbreeding of a single outbred population in Raleigh, North Carolina, USA (Mackay et al., 2012). Whole genome sequence data of all lines were downloaded from the DGRP2 website. SNPs called with a call rate of less than 95% or minor allele frequency (MAF) smaller than 0.01 and individuals with a call rate less than 95% were excluded. In total, 2,863,909 SNPs of the 185 *Drosophila* lines for which transcriptome data were also available were used for this study. Beagle 4.0 (https://faculty.washington.edu/browning/beagle/b4_0.html) was used for the imputation of missing SNP genotypes (Browning and Browning, 2013).

#### Transcriptome Data

The abundances of RNA products of 18,140 genome-wide annotated genes and novel transcribed regions (NTRs) in 185 DGRP lines was quantified using Affymetrix *Drosophila* 2.0 genome-tiling arrays, with two biological replicates for each sex. Since the correlation coefficient between the two replicates on average across all lines reached 0.95, we randomly chose one replicate for this study. The mated 3- to 5-d-old flies were collected between 1:00 and 3:00 p.m., and RNA was extracted from the flies homogenized with 1 mL of QIAzol lysis reagent (Qiagen) and two 0.25-in ceramic beads (MP Biomedical). For details on fly husbandry, RNA extraction, RNA sequence annotation and quality control see (Huang et al., 2015).

#### Phenotype Data

In total, nine traits, which were measured on females and males separately were used: startle response (STR), starvation resistance (STV), alcohol sensitivity and tolerance (AST), food intake (FI), and olfactory perceptions to five chemical odorants: olfactory perceptions to 2-Heptanone (OP2H), Methyl Salicylate (OPMS), l-Carvone (OPIC), 1-Hexanol (OP1H), Ethyl Butyrate (OPEB). These phenotypes are line means or medians of repeated measurements in different ways, and are treated as response variables in our statistical model. For startle response (starvation resistance), there were on average 40 ± 4 (52 ± 11) measurements for females, and 40 ± 4 (52 ± 11) measurements for males, the line medians were taken in several replicates for each trait (Mackay et al., 2012). The line mean of AST was calculated from two replicated measurements for each sex per line (Morozova et al., 2015). The line mean of food intake was measured from six replicate assays per sex per DGRP line (Garlapow et al., 2015). For olfactory perceptions to five chemical odorants, the average of 10 measurements was calculated as the response score of each individual trial and the averages of 10 trials on the same genotype and sex were recorded as the line means (Arya et al., 2015). The line means and variances are shown in Table 1.

**Table 1:** Line means (M) and variances (V) of phenotypes and heritability estimates for the nine traits in males and females.

	**Female**					**Male**					
**Traits**	**M**	**V**	**${\hat{H}}_{G}^{2}$**	**${\hat{H}}_{GT}^{2}$**	**${\hat{h}}_{GR}^{2}$**	**M**	**V**	**${\hat{h}}_{G}^{2}$**	**${\hat{H}}_{GT}^{2}$**	**${\hat{H}}_{GR}^{2}$**	***r***
STR	28.75 ± 0.44	40.29	0.703	0.739	0.842	28.29 ± 0.50	41.22	0.701	0.749	0.801	0.958
STV	60.61 ± 0.89	159.06	0.898	0.943	0.948	45.65 ± 0.67	90.39	0.805	0.807	0.903	0.684
AST	17.36 ± 0.28	14.03	0.943	0.944	0.972	16.49 ± 0.24	10.45	0.730	0.923	0.978	0.685
FI	0.99 ± 0.04	0.36	0.566	0.545	0.908	1.02 ± 0.05	0.50	0.989	0.988	0.980	0.674
OP2H	3.10 ± 0.04	0.28	0.819	0.823	0.840	3.04 ± 0.04	0.28	0.258	0.299	0.616	0.760
OPMS	3.40 ± 0.03	0.15	0.586	0.605	0.839	3.32 ± 0.03	0.17	0.385	0.361	0.673	0.582
OPIC	3.50 ± 0.03	0.20	0.525	0.520	0.750	3.39 ± 0.03	0.21	0.851	0.853	0.925	0.697
OP1H	2.30 ± 0.04	0.28	0.520	0.565	0.748	2.34 ± 0.04	0.28	0.362	0.536	0.635	0.794
OPEB	3.51 ± 0.03	0.18	0.462	0.673	0.848	3.57 ± 0.03	0.16	0.694	0.719	0.833	0.594

*${ĥ}_{G}^{2}$ denotes the SNP-based genomic heritability calculated with GBLUP; ${Ĥ}_{GT}^{2}$ denotes the SNP and gene expression data-based broad sense heritability calculated with GTBLUP; ${Ĥ}_{GR}^{2}$ denotes the SNP and gene expression data-based broad sense heritability calculated with GRBLUP. r denotes the phenotypic correlation between female and male phenotypes across lines*.

#### Availability of Supporting Data

The whole genome sequence data, gene expression data of 185 DGRP lines, and phenotype data of nine traits are available on *Drosophila melanogaster* Genetic Reference Panel (DGRP, http://dgrp2.gnets.ncsu.edu).

### Statistical Models

To remove the gender effect in prediction, we performed the subsequent analyses with female and male data separately. Predictions of phenotypes were done with three basic approaches and two combined methods. The basic approaches were genomic BLUP (GBLUP) to predict phenotypes using genotype data, transcriptomic BLUP (TBLUP) predicting phenotypes using transcriptome data with a linear kernel, and RKHS predicting phenotypes using transcriptome data with a Gaussian kernel (Gianola and van Kaam, 2008). The combined methods, integrating genomic and transcriptome data, were GTBLUP (combining GBLUP and TBLUP) and GRGLUP (combining GBLUP and RKHS).

#### GBLUP

As a baseline, we used SNP data of 185 *Drosophila* lines to conduct the benchmark GBLUP (VanRaden, 2008). The statistical model for GBLUP is

(1) $$\begin{array}{l} y=1\mu +\text{g}+e, \end{array}$$

where $\text{g}\sim N\left(0,G\sigma_{g}^{2}\right)$ and **e**~ $N\left(0,I\sigma_{e}^{2}\right)$ are vectors containing random breeding values and residual effects, respectively and where μ is the overall mean. The genomic relationship matrix ***G*** was calculated as $G=\frac{{\text{Z}\text{Z}}^{\prime }}{2\Sigma p_{i}\left(1-p_{i}\right)}$ (VanRaden, 2008), where *p*_*i*_ denotes the minor allele frequency (MAF) of marker *i*. Moreover, ***Z*** denotes the MAF adjusted marker matrix with entries (0−2*p*_*i*_) and (2−2*p*_*i*_) for genotypes AA and aa, respectively.

#### TBLUP

In this approach, transcriptome data of the 185 *Drosophila* lines were used as predictor variables. The statistic model is:

(2) $$\begin{array}{l} y=1\mu +t+e \end{array}$$

where $t\sim N\left(0,E\sigma_{t}^{2}\right)$ is a transcriptomic line effect. The corresponding variance-covariance matrix is ***E*** = ***RR***′ which is a linear kernel calculated from an *n* × *m* matrix ***R*** of standardized gene expression levels from *n* lines and *m* genes. The standardization of gene expression levels was conducted by calculating $r_{ij}=\frac{x_{ij}-{\bar{x}}_{j}}{s_{j}}$, where *x*_*ij*_ is the expression level of gene *j* in line *i*, ${\bar{x}}_{j}$ is the mean expression level of gene *j* across all lines, and *s*_*j*_ is the standard deviation of gene expression level of gene *j*.

#### Reproducing Kernel Hilbert Space Regression (RKHS)

Analogously, to the previously described approaches, the statistical model was:

(3) $$\begin{array}{l} y=1\mu +v+e \end{array}$$

where $v\sim N\left(0,K\sigma_{v}^{2}\right)$ is a random effect measured by transcriptome data with ***K*** being the genetic covariance matrix (Gianola et al., 2006). We chose the Gaussian kernel to calculate the genetic covariance between lines by

(4) $$\begin{array}{l} K_{ij}=k\left(r_{i},r_{j}\right)=exp\left(-\frac{||r_{i}-r_{j}|{|}^{2}}{h}\right) \end{array}$$

Here, *h* is a bandwidth parameter, which controls how fast the covariance function drops as points get further apart. The vector *r*_*i*_ gives the vector of standardized expression levels of line *i* across all genes, and *r*_*j*_ is the vector of standardized expression levels of line j across all genes. The bandwidth parameter *h* was chosen using a grid search approach under cross-validation, aiming at finding a suitable value that maximized the predictive correlation within a model setting (Jones et al., 1996; Gianola and Schön, 2016).

#### GTBLUP

In GTBLUP, transcriptome data was integrated into genomic prediction. SNP data and transcriptome data of 185 *Drosophila* lines were treated as predictor variables. The prediction model was:

(5) $$\begin{array}{l} y=1\mu +\text{g}+t+e \end{array}$$

where all variables are defined as described above.

#### GRBLUP

The statistical model for GRBLUP can be expressed as

(6) $$\begin{array}{l} y=1\mu +g+v+e \end{array}$$

The only difference between GTBLUP and GRBLUP is that in GRBLUP we replace $t\sim N\left(0,E\sigma_{t}^{2}\right)$ of GTBLUP with $v\sim N\left(0,K\sigma_{v}^{2}\right)$ of RKHS. Again ***K*** is the genetic covariance matrix constructed by the Gaussian kernel (4) and the optimum bandwidth parameter h is found by grid-search and cross-validation.

### Estimation of the Omics-Augmented Broad Sense Heritability Based on the Between Line Effects

The omics-augmented broad sense heritability was defined as the proportion of phenotypic variance explained by whole genome SNP markers and other omics data,

(7) $$\begin{array}{l} {Ĥ}_{o}^{2}=\frac{{\hat{\sigma }}_{g}^{2}+{\hat{\sigma }}_{omics}^{2}}{{\hat{\sigma }}_{g}^{2}+{\hat{\sigma }}_{omics}^{2}+{\hat{\sigma }}_{e}^{2}} \end{array}$$

where ${\hat{\sigma }}_{\text{g}}^{2}$ denotes the proportion of additive genetic variance explained by the whole genome SNP markers and ${\hat{\sigma }}_{omics}^{2}$ denotes the variances explained by one or several omics data layers which can be the transcriptome, proteome, metabolome, epigenome, metagenome etc.

(1) SNP-based genomic narrow sense heritability for GBLUP (${ĥ}_{G}^{2}$).

The SNP-based genomic narrow sense heritability is defined as the proportion of phenotypic variance explained by SNP marker effects. This SNP-based heritability is calculated as

(8) $$\begin{array}{l} {ĥ}_{G}^{2}=\frac{{\hat{\sigma }}_{g}^{2}}{{\hat{\sigma }}_{g}^{2}+{\hat{\sigma }}_{e}^{2}} \end{array}$$

(2) SNP and gene expression data-augmented broad sense heritability for GTBLUP (${Ĥ}_{GT}^{2}$) and GRBLUP (${Ĥ}_{GR}^{2}$)

The proportion of phenotypic variance explained by SNP data and gene expression data in GTBLUP (${Ĥ}_{GT}^{2}$) is calculated as

(9) $$\begin{array}{l} {Ĥ}_{GT}^{2}=\frac{{\hat{\sigma }}_{g}^{2}+{\hat{\sigma }}_{t}^{2}}{{\hat{\sigma }}_{g}^{2}+{\hat{\sigma }}_{t}^{2}+{\hat{\sigma }}_{e}^{2}} \end{array}$$

and in GRBLUP (${Ĥ}_{GR}^{2}$) are calculated as

(10) $$\begin{array}{l} {Ĥ}_{GR}^{2}=\frac{{\hat{\sigma }}_{g}^{2}+{\hat{\sigma }}_{v}^{2}}{{\hat{\sigma }}_{g}^{2}+{\hat{\sigma }}_{v}^{2}+{\hat{\sigma }}_{e}^{2}} \end{array}$$

The variance components ${\hat{\sigma }}_{g}^{2}$, ${\hat{\sigma }}_{t}^{2}$, ${\hat{\sigma }}_{v}^{2}$, ${\hat{\sigma }}_{e}^{2}$ from models (1), (5), and (6) were estimated from the entire data sets, using the R package “regress” (Clifford and McCullagh, 2014), which also provided predictions of random effects.

### Comparison of Predictive Abilities

The different approaches were assessed using 20 replicates of a 5-fold cross-validation (Erbe et al., 2013). Predictive abilities were defined as the Pearson's correlation coefficients between predicted genetic values and observed phenotypes in the test sets. The final predictive ability of each model was the mean of the predictive abilities across 100 estimates. Overall predictive abilities among the five models implemented in the study were compared using a Tukey's honest significant difference test (Tukey, 1949).

## Results

### Estimation of “Omics-Augmented Broad Sense Heritability” Based on the Between Line Effects and Variance Components

**Figure 1:**
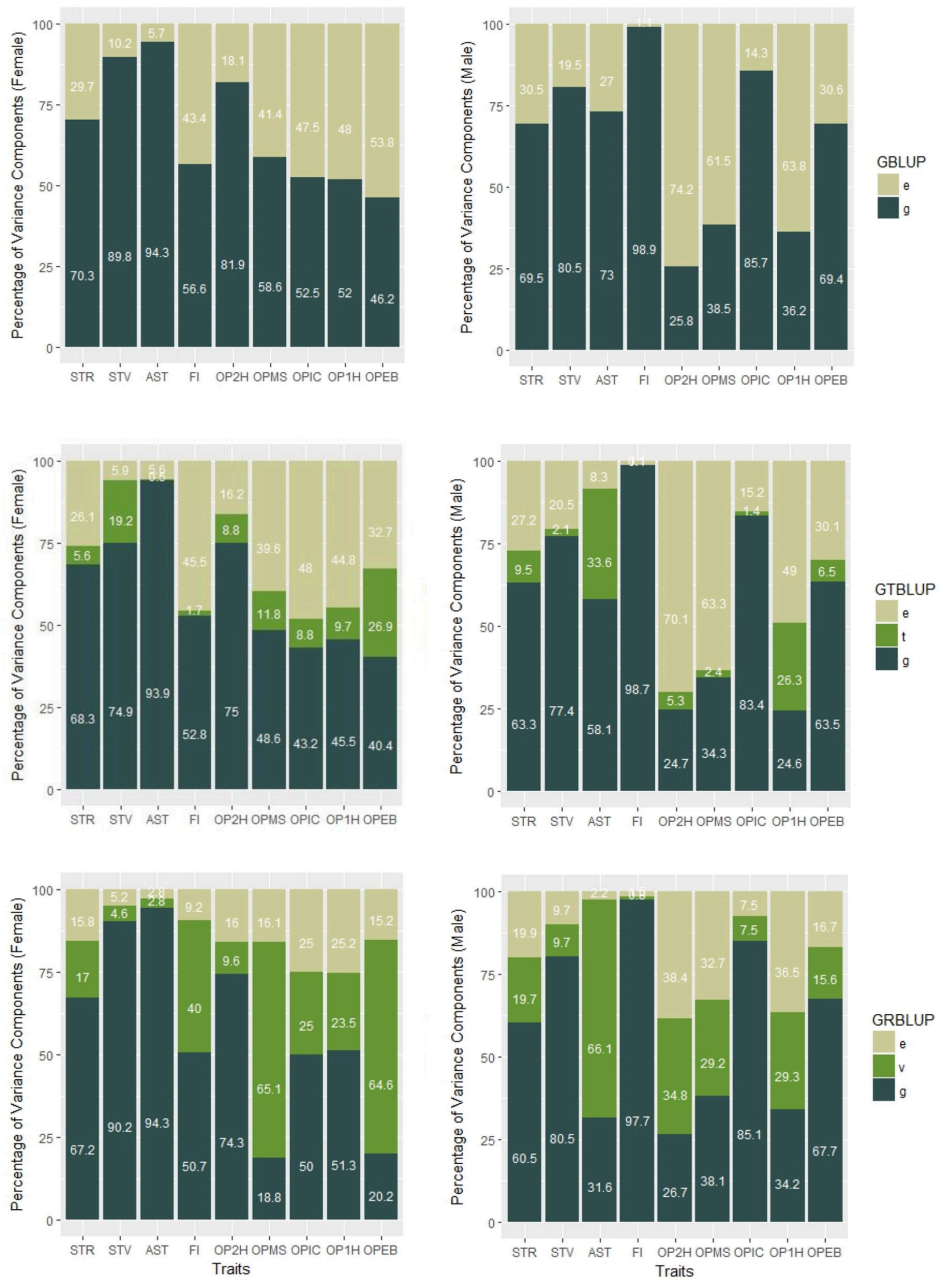
Percentages of variance components of GBLUP, GTBLUP, and GRBLUP for nine traits for females **(left)** and males **(right)**. e is the residual; t is the transcriptomic line effect in GTBLUP; v is the transcriptomic line effect in GRBLUP, and g is the additive genetic effect captured by SNP data.

Genomic heritabilities obtained with model (1) ranged from 0.25 to 0.99 and are generally high. On average across all traits, they are slightly higher for females (${ĥ}_{Gf}^{2}=0.66\;\pm 0.059\;)$ than for males (${ĥ}_{Gm}^{2}=0.63\;\pm 0.081)$ (see Figure 1 and Table 1). It should be noted, though, that these values pertain to the average performance of many replications of inbred individuals, and thus should not be compared to narrow sense heritability estimates on an individual base.

In GTBLUP and GRBLUP, we integrated transcriptome data into genomic prediction. The only difference between these two methods is that two different kernels were used to construct the relationship matrix based on transcriptome data. For the SNP and gene expression data-augmented heritability, ${Ĥ}_{GR}^{2}$ was higher than ${Ĥ}_{GT}^{2}$ for almost all traits and in both sexes (Table 1). Only the trait FI did not show this pattern for males. Across all traits, ${Ĥ}_{GR}^{2}$ had a mean of 0.85 ± 0.050 for females and 0.81 ± 0.080 for males compared to ${Ĥ}_{GT}^{2}$ 0.71 ± 0.025 for females, and 0.69 ± 0.049 for males. Compared to GTBLUP, GRBLUP captured more genetic variance explained by gene expression data for some traits, especially for some traits with relatively low SNP-based genomic heritability $h_{G}^{2}$, such as FI, OPMS, OPIC, OP1H, and OPEB in females and AST, OP2H, OPMS, and OP2H in males.

### Overall Predictive Ability

**Figure 2:**
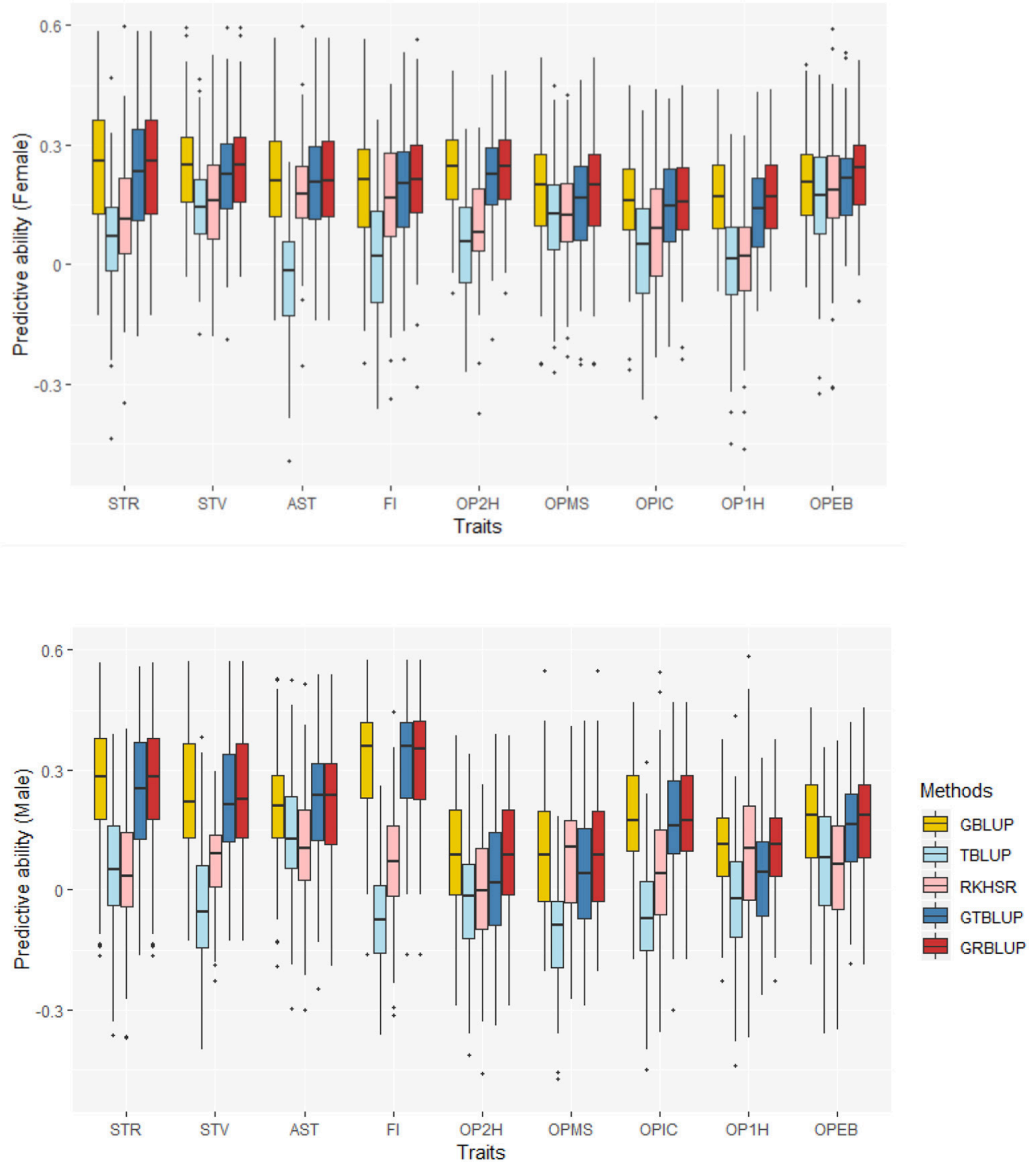
Predictive abilities for nine traits with five statistical models in females and males.

The predictive abilities of the nine traits obtained with the 5 statistical models for females and males are shown in Figure 2 and Supplementary Table 1. GBLUP as the reference method provided predictive abilities ranging from 0.162 ± 0.012 to 0.240 ± 0.013 in females and from 0.095 ± 0.015 to 0.325 ± 0.013 in males across all traits. For GBLUP, the proportion of phenotypic variance explained by SNP data and genomic predictive abilities were highly positively correlated. The correlation coefficients were 0.731 and 0.885 for females and males, respectively. Transcriptome-based prediction alone was not accurate for most traits: observed predictive abilities were 0.001 ± 0.013 to 0.182 ± 0.011 for females, and 0.036 ± 0.014 to 0.107 ± 0.014 for males with RKHS and −0.035 ± 0.011 to 0.165 ± 0.014 for females and −0.113 ± 0.013 to 0.13 ± 0.015 for males with TBLUP. The correlation between female and male predictive abilities with RKHS and TBLUP were low with correlation coefficients of 0.077 and −0.189, respectively.

**Figure 3:**
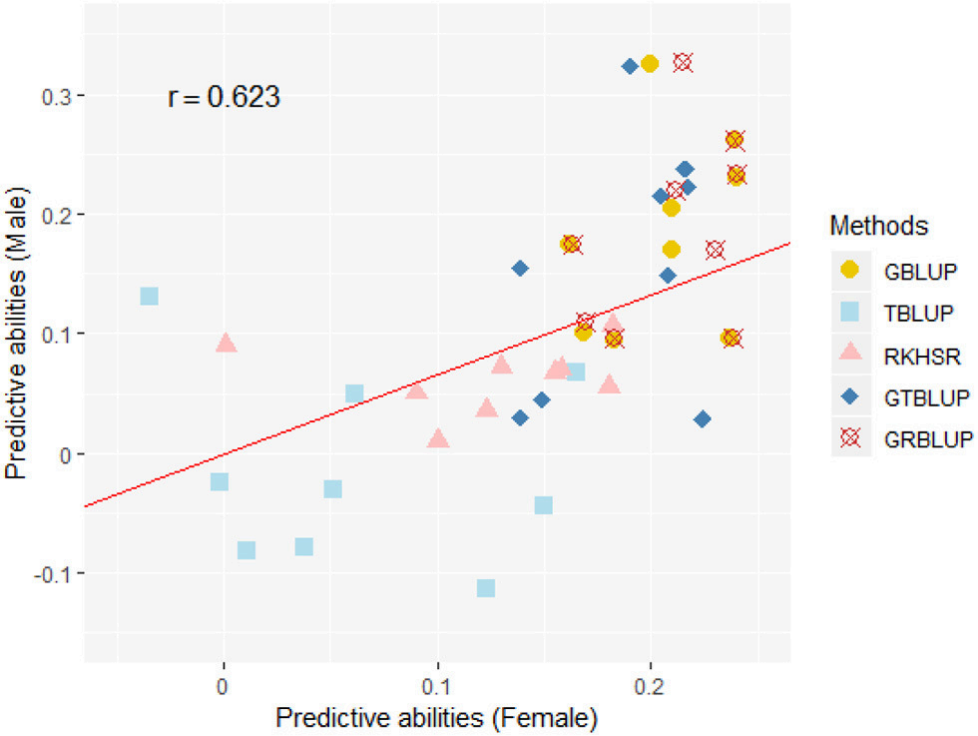
The correlation between predictive abilities in females and males across nine traits and five statistical models. r denotes the Pearson correlation coefficient between female and male predictive abilities across all traits and all statistical models. The red line denotes a standardized major axis regression line.

**Figure 4:**
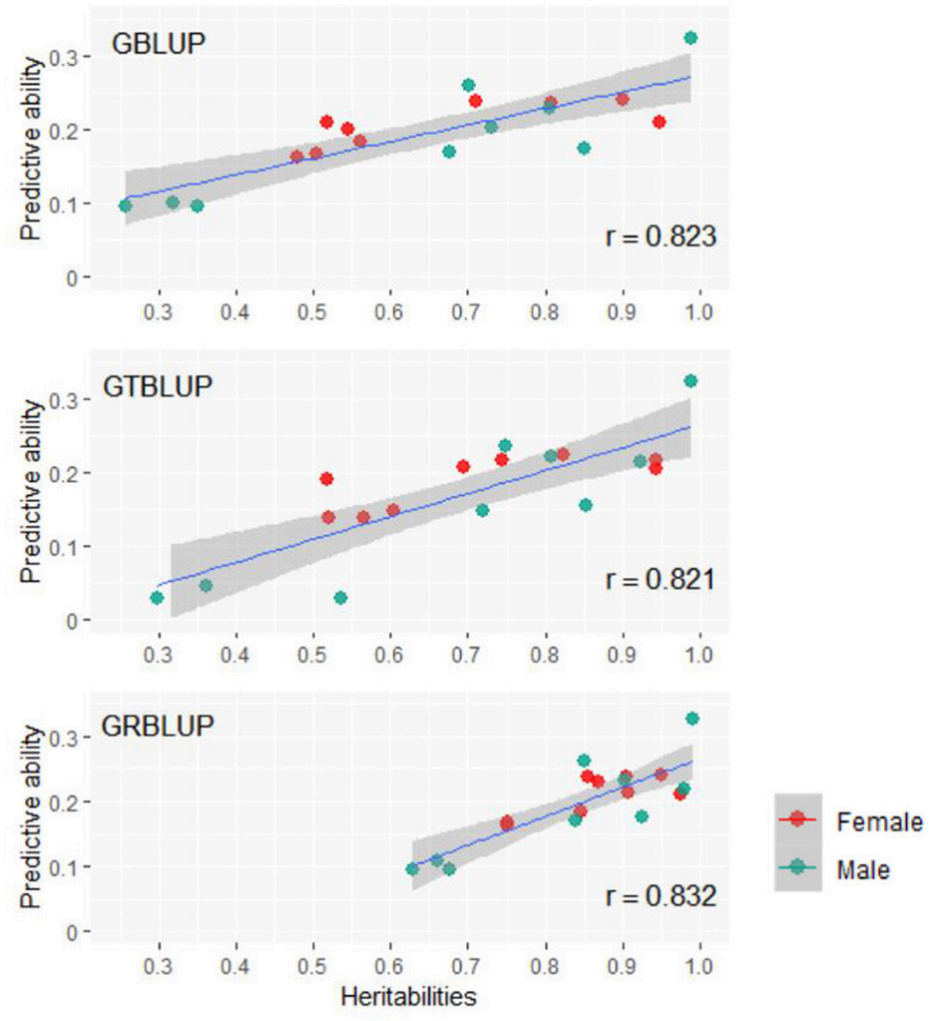
The correlation between heritabilities ${ĥ}_{G}^{2}$, ${Ĥ}_{GT}^{2}$, ${Ĥ}_{GR}^{2},$ and predictive abilities for GBLUP, GTBLUP, and GRBLUP across all traits and both sexes. *r* denotes the Pearson correlation coefficient. The blue lines denote the overall linear regression lines. The gray shadow denotes the 0.95 confidence interval.

Except for one trait (OPEB) in females, there was no significant difference of predictive abilities between GRBLUP and GBLUP. For the trait OPEB in female, GRBLUP (0.23 ± 0.012) gave a higher predictive ability than GBLUP (0.208 ± 0.012). Both GRBLUP (female 0.21, male 0.187) and GBLUP (female 0.205, male 0.184) provided better predictive abilities on average in all traits than GTBLUP (female 0.187, male 0.156) for female and male. It is worth noting that predictive abilities between males and females for all models were found to be remarkably different for six out of nine traits (AST, FI, OP2H, OPMS, OPIC, OP1H). In females, the predictive abilities of three models (GBLUP, GTBLUP and GRBLUP) varied slightly among all nine traits with a range between 0.139 ± 0.012 (OP1H in GTBLUP) and 0.24 ± 0.013 (STV in GRBLUP), while in males the predictive abilities of these three models have a more significant variation ranging from 0.045 ± 0.014 (OPMS in GTBLUP) to 0.326 ± 0.014 (FI in GRBLUP). The correlation coefficient between predictive abilities in females and males across all traits and models is 0.623 (Figure 3). The correlation coefficients between heritabilities ${ĥ}_{G}^{2}$, ${Ĥ}_{GT}^{2}$, ${Ĥ}_{GR}^{2}$ and predictive abilities for GBLUP, GTBLUP, GRBLUP across all traits and both sexes are 0.823, 0.821, and 0.832 respectively (Figure 4). The bandwidth parameter h in the Gaussian kernel varied dramatically from 0.7 to 270,000, and choosing the right value had great impact on predictive abilities of RKHS and GRBLUP.

## Discussion

Previous *Drosophila* genomic prediction studies have shown that there is a high degree of genotype by sex interaction in some traits. Ober et al. (2012) showed that given the significant sex by line interaction variance in starvation resistance, the prediction is more accurate in females than in males (0.254 vs. 0.203), and in chill coma recovery time the predictive ability is very low for female and zero for male. It has also been found that 42% of the *Drosophila* transcriptome is genetically variable between males and females, including the NTRs (Huang et al., 2015). We also found expression patterns to be clearly separated between males and females (see Supplementary Figure 1) and thus we performed all analyses on females and males separately in order to remove the gender effect in prediction.

### Omics-Augmented Broad Sense Heritability

Yang et al. (2010) showed that 45% of the variance for human height can be explained by considering all SNPs simultaneously when using GBLUP to estimate the narrow sense heritability, the proportion of phenotypic variance due to additive genetic variance. Two explanations for the “missing heritability” were provided: (1) the causal variants each explain such a small amount of variation that their effects do not reach stringent significance thresholds, or (2) the causal variants are not in complete linkage disequilibrium (LD) with the SNPs that have been genotyped. Speed et al. (2012) argued that GBLUP may not be capable to provide unbiased estimates of the genomic heritability, and a main reason is that in the computation of the G matrix the LD between SNPs and QTL is ignored. Kim et al. (2017) proposed that the main problem of estimating genomic heritability does not reside in the manner the G matrix is computed, but rather in the use of massive numbers of markers that are in LD with QTL. Since there is probably no complete linkage disequilibrium between SNPs and all causal variants, which e.g., also can be structural variants, using SNP data may not provide accurate estimates of narrow sense heritability. Narrow sense heritability estimates play a key role in predicting or assessing the effectiveness of artificial selection in that they provide a way to measure the extent to which additive genetic variance is related to phenotypic variance in a specific population (Visscher et al., 2008). However, for the prediction of phenotypes, the concept of broad sense heritability is more useful than the concept of narrow sense heritability, because it reflects all the genetic contributions to a population's phenotypic variance including additive and non-additive effects, which provides upper limits to estimates of transmissible genetic variance (Lush, 1940; Stoltenberg, 1997). Nevertheless, as mentioned, even if all SNPs were used, only part of the genetic effects can be captured. The inclusion of additional layers of genomic information in the prediction machinery may provide a partial solution for this problem. When DNA information is transcribed into RNA and then expressed as protein products, abundance of gene expression products is one of the intermediate layers in this process. We assume that the missing additive variance in estimation of narrow sense heritability by using SNP data, and some non-additive effects may be captured by the gene expression data. In this case, utilizing both SNP data and gene expression data to estimate broad sense heritability can be a promising approach. The classical definition of broad sense heritability is the ratio of genetic variance to the phenotypic variance, which implicitly assumes that all genetic variation must be encoded at the genome level. However, gene expression data may be inevitably affected by some external regulation which belongs to environment effects in terms of the classical genetic model, where the phenotype is considered to be affected by genetic and environmental effects, and the interaction between both. In the multi-omics era, the input information for the phenotypic prediction machinery is not restricted to gene or genome layer. Multi-omics data reflecting the transcriptome, proteome, metabolome, epigenome, metagenome etc. are increasingly exploited as input data for the phenotypic prediction (Acharjee et al., 2016; Xu et al., 2016). Thus, we discuss the concept “omics-augmented broad sense heritability” for the prediction of phenotype which not only includes the effects at the genome level (both additive and non-additive), but also includes the effects of downstream biological regulation captured by one or several omics layers. In phenotype prediction this concept can help to measure the extent to which the information in the different layers of multi-omics data is related to phenotypic variance in a specific population. For some traits substantially affected by non-additivity and downstream biological regulation effects, or with poor LD between SNPs and QTL, the estimated genomic heritabilities may be low so that they may be inadequate as a measure of predictive ability. In this case the omics-based broad sense heritability may be more informative than narrow or broad sense heritability because of the inclusion of non-additive effects and biological regulation effects in the numerator of${\;Ĥ}_{o}^{2}$, and it can be seen as the potential upper limit of the predictive ability of phenotypic prediction when utilizing multi-omics data. This method was used to measure the increased heritabilities of 11 traits when incorporating gene expression and metabolic data into phenotypic prediction in maize, however, without discussing the reasonability (Guo et al., 2016). It must be highlighted that the “omics—augmented broad sense heritability” is just available in the context of phenotype prediction, while in the genomic prediction for breeding values this concept is of limited usefulness because the biological regulation variance in the numerator of ${Ĥ}_{o}^{2}$ is not fully heritable. The approach should be seen as a complement or partial substitution to the classical narrow sense heritability when using multi-omics data to predict phenotypes.

### Assessment of Predictive Abilities

Due to the transmission of genetic information from DNA sequence to transcripts, information at the gene expression layer (transcriptome) is “closer” to phenotypes than genomic information, and thus should help providing better predictions of phenotypes than genomic information. However, unlike the DNA sequence, the transcriptome information is not stably inherited and measurements of transcriptome abundance are affected by choice of tissue, time of sampling and experimental conditions. In this study, predictive abilities of RKHS obtained on 9 traits were relatively low (0.001 to 0.182 in female, 0.036 to 0.107 in male), and were much lower than predictive abilities obtained with GBLUP using SNP data. A similar result was also shown in maize, where predictive abilities of transcriptomic prediction were always lower than the genomic prediction when comparing both using eight statistical models (Xu et al., 2017). RKHS and GRBLUP performed significantly better than TBLUP and GTBLUP, indicating that RKHS with a Gaussian should be preferred when conducting transcriptome-based prediction.

For GBLUP, we found predictive ability and the phenotypic variance component explained by SNP data to be highly positively correlated with correlation coefficients of 0.73 and 0.89 for females and males, respectively. However, the phenotypic variance explained by SNP data was exceedingly high (>0.8) for some traits, such as STV, AST, OP2H in females and STV, AST, FI, OPIC in males, while the predictive abilities for these traits were relatively low. The reason could be the small sample size of lines and this result was consistent with the previous study for starvation resistance and startle response which the predictive abilities were 0.239 ± 0.012 and 0.23 ± 0.012, respectively. Ober et al. (2012) showed that the predictive ability could reach 0.58 if the number of sequenced lines for training was increased to 1,000 (Ober et al., 2012).

We incorporated transcriptome data with genomic prediction using GRBLUP which combine the standard GBLUP and the RKHS method. From an RKHS point of view, the genomic relationship matrix G in GBLUP can be viewed as a parametric kernel that only captures genetic values based on an additive genetic relationship among individuals. The Gaussian kernel is a non-parametric kernel which may pick up genetic signals regardless of the underlying genetic architecture. Choosing the most suitable bandwidth parameter h can provide an optimal $\frac{\sigma_{k}^{2}}{\sigma_{k}^{2}+\sigma_{e}^{2}}$ ratio, which gives an appropriate weight to the phenotypic variance explained by transcriptome data, leading to an optimized predictive performance. GRBLUP can be considered as a case of RKHS with two kernels. For the comparison between GTBLUP and GRBLUP, the only difference between these two methods is that two different kernels were used to construct a relationship matrix based on transcriptome data. In GTBLUP, we replaced the Gaussian kernel used in GRBLUP with a linear kernel. Compared with GBLUP, the SNP and gene expression data-based broad sense heritability $H_{GT}^{2}$ of GTBLUP was higher than the SNP-based genomic heritability $h_{G}^{2}$ of GBLUP at all 9 traits in both male and female, but GTBLUP slightly decreased the combined predictive ability for most traits. This result suggests that there may be an overfitting problem when using GTBLUP to model the combined data. Xu et al. (2017) observed an analogical result which decreased the predictive ability when combining transcriptome data and metabolic data into genomic prediction for six yield-related traits in maize. Compared to GTBLUP, GRBLUP captured more genetic variance explained by gene expression data for some traits, especially for traits with relatively lower genomic heritability $h_{G}^{2}$ in GBLUP, such as FI, OPMS, OPIC, OP1H, OPEB in female; and AST, OP2H, OPMS, OP2H in male. For the omics-based broad sense heritability based on the between line effects, ${Ĥ}_{GR}^{2}$ was higher than ${Ĥ}_{GT}^{2}$ for all 9 traits in both males and females, and GRBLUP provided a superior predictive ability than GTBLUP across all traits. This demonstrated that the Gaussian kernel is superior to the linear kernel *E* = *RR*^*T*^ for modeling transcriptome data in genomic prediction.

In our result, there was only one trait (OPEB in females) for which the predictive ability of GRBLUP (0.23) was higher than the predictive ability of GBLUP (0.21). This indicated that predictive ability can be improved when combining transcripts with SNPs using GRBLUP, but it depends on the traits. For the rest of the traits for both males and females, the SNP and gene expression data-based heritability $H_{GR}^{2}$ was remarkably increased compared to the SNP-based heritability $h_{G}^{2}$ of GBLUP. However, there is no significant difference in predictive ability between GRBLUP and GBLUP, which might be caused by the small sample size and may be changing with increased sample sizes.

## Conclusion

We constructed a semiparametric prediction model (GRBLUP) with two kernels combining SNP and transcriptome data. The parametric G kernel was used to capture the additive genetic part, and the Gaussian kernel is a non-parametric kernel which was used to pick up non-additive genetic effects and biological regulation effects regardless of the underlying genetic architecture. In our study, GRBLUP and GBLUP provided similar predictive ability, but GRBLUP could capture more phenotypic variance components explained by transcriptome data. The better goodness of fit of GRBLUP in general did not translate into a better predictive ability. It should be noted, though, that sample size was small and gene expression was not measured at one time point and in one specific tissue functionally linked to the trait of interest. However, including transcriptomic data can increase predictive ability, as was shown for the trait OLED in females. We conclude that adding more specifically collected transcriptome data has the potential to improve genomic predictions in larger scale applications.

## Author Contributions

All authors were involved in the design of the study. ZL performed the model validations and wrote the manuscript, with contributions of HS. NG and JM participated in discussing the statistical models. All authors commented on the manuscript and read and approved the final version.

### Conflict of Interest Statement

The authors declare that the research was conducted in the absence of any commercial or financial relationships that could be construed as a potential conflict of interest.
