# Supplementary material for: Integrating Gene Expression Data Into Genomic Prediction
Source: Front Genet. 2019 Feb 25;10:126. doi: 10.3389/fgene.2019.00126 (PMC6397893; doi:10.3389/fgene.2019.00126)

Supplementary Figure 1: PC analysis of female (red) and male lines (green) for gene expression data. The variances explained by PC 1 (x-axis) and PC 2 (y-axis) are shown in the respective captions.

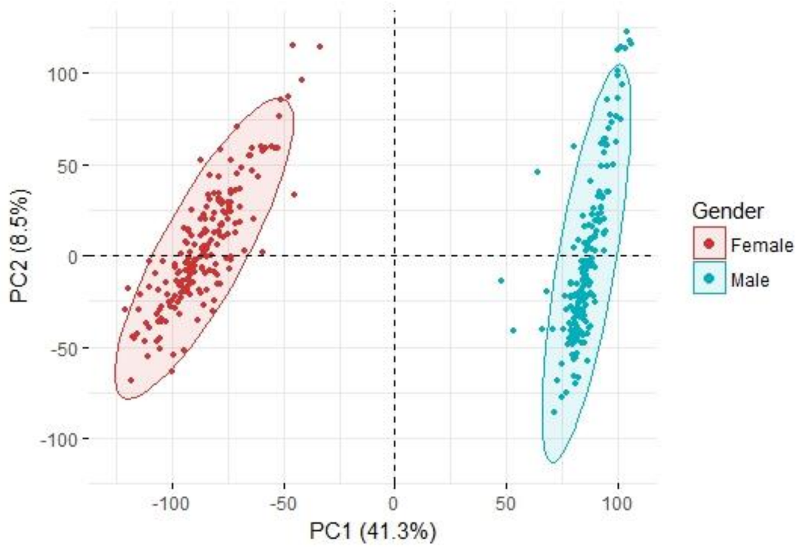

Supplement: Supplementary file 1 [file Image_1.pdf]
