# Supplementary material for: Integrating Gene Expression Data Into Genomic Prediction
Source: Front Genet. 2019 Feb 25;10:126. doi: 10.3389/fgene.2019.00126 (PMC6397893; doi:10.3389/fgene.2019.00126)
Supplement: Supplementary file 2 [file Table_1.pdf]

supplementary material

Supplementary table 1: Empirical prediction accuracy ± standard deviation of 9 traits in 5 statistical models for both females and males.

	Female					Male				
**Traits**	GBLUP	TBLUP	RKHS	GTBLUP	GRBLUP	GBLUP	TBLUP	RKHS	GTBLUP	GRBLUP
**STR**	0.239 ± 0.015	0.061 ± 0.013	0.123 ± 0.014	0.216 ± 0.014	0.239 ± 0 .015	0.261 ± 0.015	0.049 ± 0.014	0.036 ± 0.014	0.237 ± 0.016	0.261 ± 0.015
**STV**	0.240 ± 0.013	0.150 ± 0.014	0.155 ± 0.013	0.217 ± 0.013	0.240 ± 0.013	0.230 ± 0.014	-0.044 ± 0.013	0.067 ± 0.011	0.222 ± 0.014	0.233 ± 0.014
**AST**	0.210 ± 0.013	-0.035 ± 0.011	0.182 ± 0.011	0.205 ± 0.013	0.211 ± 0.013	0.204 ± 0.014	0.130 ± 0.015	0.107 ± 0.014	0.215 ± 0.015	0.220 ± 0.015
**FI**	0.200 ± 0.015	0.011 ± 0.015	0.158 ± 0.016	0.190 ± 0.015	0.215 ± 0.014	0.325 ± 0.013	-0.081 ± 0.015	0.070 ± 0.013	0.324 ± 0.013	0.326 ± 0.014
**OP2H**	0.237 ± 0.012	0.051 ± 0.012	0.100 ± 0.011	0.224 ± 0.012	0.238 ± 0.012	0.096 ± 0.014	-0.030 ± 0.013	0.010 ± 0.013	0.029 ± 0.016	0.096 ± 0.014
**OPMS**	0.183 ± 0.015	0.123 ± 0.015	0.130 ± 0.013	0.149 ± 0.013	0.183 ± 0.015	0.095 ± 0.015	-0.113 ± 0.013	0.072 ± 0.014	0.045 ± 0.014	0.096 ± 0.015
**OPIC**	0.162 ± 0.012	0.038 ± 0.015	0.090 ± 0.015	0.139 ± 0.013	0.163 ± 0.012	0.175 ± 0.014	-0.078 ± 0.011	0.050 ± 0.015	0.155 ± 0.015	0.175 ± 0.014
**OP1H**	0.168 ± 0.011	-0.002 ± 0.013	0.001 ± 0.013	0.139 ± 0.012	0.169 ± 0.011	0.100 ± 0.012	-0.025 ± 0.011	0.090 ± 0.015	0.030 ± 0.012	0.110 ± 0.012
**OPEB**	0.210 ± 0.012	0.165 ± 0.014	0.180 ± 0.014	0.208 ± 0.011	0.230 ± 0.012	0.170 ± 0.013	0.068 ± 0.015	0.056 ± 0.014	0.149 ± 0.012	0.170 ± 0.013
